# Monitoring outcomes of substance use disorder among healthcare professionals in Utah: A retrospective study of the professionals health program

**DOI:** 10.1111/ajad.70106

**Published:** 2025-12-16

**Authors:** Mubo O. Olufemi, Lisa J. Merlo, Melissa Cheng, Hongwei Zhao, Justin Yang, Matthew S. Thiese

**Affiliations:** ^1^ Department of Family and Preventive Medicine Rocky Mountain Center for Occupational and Environmental Health, School of Medicine, University of Utah Salt Lake City Utah USA; ^2^ Department of Psychiatry University of Florida Gainesville Florida USA; ^3^ Environmental and Occupational Medicine and Epidemiology (EOME) Program, Department of Environmental Health Harvard T.H. Chan School of Public Health Boston Massachusetts USA; ^4^ Department of General Internal Medicine Boston University Chobanian & Avedisian School of Medicine Boston Massachusetts USA

## Abstract

**Background and Objectives:**

Substance use disorders (SUD) among healthcare professionals threaten both patient safety and workforce stability. Professional health programs (PHPs) aim to support recovery and safe practice reentry, yet outcomes for non‐physician professionals remain underexplored. This study assessed return to use and professional outcomes among healthcare professionals monitored through the Utah Professionals Health Program (UPHP), a cohort predominantly composed of non‐physician participants.

**Methods:**

A retrospective cohort study used data from 183 UPHP participants with SUD (60.7% nurses) enrolled between 2013 and 2024. The primary outcome was return to use, defined by positive toxicology results or self‐report. Logistic regression was used to identify predictors of return to use.

**Results:**

Return to use occurred in 48.6% of participants, with the highest incidence (33.9%) in year one. By year five, 81.4% had completed monitoring, and 55.9% were working full‐time in healthcare. In adjusted models, age (OR = 0.93 per year, *p* = .039), duration of SUD before UPHP enrollment (OR = 1.14 per year, *p* = .004), and injury history (OR = 0.23, *p* = .017) were significantly associated with return to use.

**Discussion and Conclusions:**

This non‐physician dominant cohort highlights need for early recovery support and profession‐specific care. Return to use was most frequent in year one, underscoring the need for intensive early‐phase monitoring. Tailored support for injury‐related substance use, along with peer support and employment services may improve outcomes.

**Scientific Significance:**

Findings provide novel insight into recovery outcomes for non‐physician healthcare professionals in a PHP, a group underrepresented in addiction research.

## INTRODUCTION

Substance use disorders (SUD) can occur in any profession, but healthcare professionals (HCPs) face especially high stakes due to the critical nature of their roles and responsibility for patient safety.^1^ Even a single instance of impairment can compromise both patient outcomes and professional integrity. Unique occupational stressors of healthcare, including long shifts, access to prescription drugs, and high‐pressure decision‐making can significantly increase the risk of substance misuse.^2^, ^3^, ^4^ The personal and professional repercussions of SUD among HCPs may be extensive, as individuals affected by SUD often suffer physical and mental health deterioration, strained interpersonal relationships, and professional setbacks.^3^ Moreover, SUD among HCPs can lead to clinical errors, diminished quality of care, and compromised public health.^5^, ^6^, ^7^, ^8^

Existing research shows that SUD among HCPs follow both shared and profession‐specific patterns. Baldisseri estimated that 10%–15% of HCPs misuse substances during their careers, noting especially high risk in demanding specialties such as anesthesia and emergency medicine.^9^ Comparative work by Shaw et al. found that nurses and physicians differ in relapse triggers, treatment experiences, and work‐related sanctions, suggesting that professional role may shape recovery outcomes.^10^

To address challenges associated with SUD in HCPs, Physician Health Programs (PHP) have been implemented nationwide, offering confidential monitoring and support for physicians (and sometimes other HCPs) with potentially impairing conditions. The Federation of State Physician Health Programs (FSPHP), is a national organization supporting state‐level PHPs in their mission to protect both HCPs and their patients.^11^ PHPs typically coordinate comprehensive assessments, facilitate access to appropriate treatment, and provide long‐term health monitoring under structured, non‐disciplinary conditions.^12^

The Utah Professionals Health Program (UPHP) mirrors this national model and establishes SUD monitoring contracts with its participants. UPHP is tailored to the needs of Utah's broader clinical workforce, including physicians, nurses, pharmacists, dentists, mental health providers, physician assistants, veterinarians, podiatrists, optometrists, and chiropractors. UPHP does not provide treatment directly; instead, it coordinates referrals, facilitates employment reentry, and monitors compliance with requirements designed to support long‐term recovery. Its structure prioritizes confidentiality: participants who adhere to the monitoring agreement avoid disciplinary reporting to licensing boards and retain privacy throughout the duration of their monitoring.^13^ The UPHP process begins with a referral, followed by a comprehensive intake meeting. Participants are then referred for a formal evaluation, which determines individualized treatment recommendations. Once the initial treatment phase is completed, participants enter a confidential monitoring program, typically lasting 5 years, which generally includes random toxicology testing, psychotherapy, regular evaluations, and participation in mutual support groups. UPHP also offers advocacy services to aid reentry to the clinical workforce and restoration of professional credentialing, as needed.^13^

Several studies have examined outcomes among professionals participating in these programs. Smiley and Reneau found factors linked to successful completion among more than 7700 nurses, while McLellan et al. and DuPont et al. reported high 5‐year recovery and return‐to‐practice rates among physicians in state PHPs.^12^, ^14^, ^15^ Despite the growing support for PHPs,^16^ there are limited data on long‐term outcomes for non‐physician professionals, particularly within individual state programs such as UPHP. Understanding return‐to‐use patterns, program completion rates, and trends in return‐to‐work is critical to ongoing efforts to improve and disseminate this model. The current study addresses this gap by conducting an 11‐year retrospective analysis of UPHP participant experiences.

## METHODS

### Study design and data collection

We conducted a retrospective cohort analysis by reviewing participant records enrolled in the UPHP and its predecessor, the Utah Recovery Assistance Program (URAP), from January 1, 2013, to December 31, 2024. UPHP was established on May 1, 2020; therefore, data prior to this date reflect URAP participants. URAP to UPHP changes were administrative in nature and did not alter toxicology criteria or definitions.

The UPHP program manager was initially contacted by the first author through a professional connection to facilitate data access. The University of Utah IRB granted exemption (Category 4; Protocol #00182728), and a confidentiality agreement was established with UPHP.

All available clinical and laboratory records, including demographics, progress notes, self‐reports, and toxicology results were extracted between October 2024 and February 2025 using Research Electronic Data Capture (REDCap)^17^, ^18^ to ensure consistency.

### Inclusion and exclusion criteria

All available UPHP participant records from the designated study period were examined. Records were excluded from analysis if they met any of the following criteria:

- Lacked essential information.
- Were illegible or too inconsistent to reliably interpret.
- Reflected duplicate enrollments; in such cases, only the most recent monitoring encounter was included in the analysis.
- Individuals evaluated but did not participate in monitoring with the UPHP.

### Data extraction and confidentiality

Data collection was conducted by the first author and subsequently reviewed by the UPHP program manager to ensure accuracy. No identifiable data were collected.

Age was recorded at contract signing. Trauma included physical, emotional, or sexual trauma. Psychiatric comorbidity reflected clinician‐diagnosed conditions. Injury/pain history captured documented musculoskeletal or chronic pain; missing data coded “unknown”.

### Outcome measures

The primary outcome was any return to substance use during the 5‐year monitoring period, defined as either a single lapse or sustained relapse, as identified via verified toxicology testing results or self‐report. Toxicology testing methods included urine drug screens, hair and fingernail analysis, blood phosphatidylethanol (PEth) testing, and remote breath‐based testing systems (e.g., Soberlink). Results were classified as: negative, prescription‐positive (i.e., positive test result for a known, approved substance such as prescription pain medication following surgery), positive for unapproved substances, or flagged for aberrancy (e.g., dilute or adulterated urine sample). Only positive results for unapproved or non‐prescribed substances, or confirmed self‐reports of use, were coded as a return to use.

Secondary outcomes included licensure and employment status at 1 and 5 years, and program completion. These intervals were selected because first‐year data were available for all cohorts (2013–2024) and year‐five data corresponded to the standard program completion point. Participants enrolled between 2013 and 2019 had complete 5‐year data and were included in all analyses; others were only included in analyses of baseline characteristics and first‐year outcomes.

### Statistical analysis

Descriptive statistics were used to summarize demographic and outcome variables. Categorical variables were compared using Chi‐square or Fisher's exact tests, as appropriate, based on expected cell counts. Return to use was modeled as a binary outcome using logistic regression, restricted to participants with complete 5‐year follow‐up data.

Univariate logistic regression models were first run for each independent variable. Candidate variables with *p* < 0.20 or strong clinical relevance were entered into the initial multivariable model: gender, age at enrollment, profession, primary substance, psychiatric comorbidities, injury history, family history of SUD or mental illness, duration of substance use, pharmacologic treatment (MAT), and dilute or aberrant toxicology test results. Given the limited sample size, a stepwise reduction approach was used to reduce the number of predictors in the final model. Covariates with the highest *p*‐values were sequentially removed, provided the Akaike Information Criterion (AIC) continued to decrease, until six predictors remained: age at enrollment, duration of substance use, injury history, gender, profession, and dilute or aberrant toxicology results. Some clinically‐relevant but statistically non‐significant variables were retained to examine their potential associations with the outcome variable. Adjusted odds ratios (ORs) and 95% confidence intervals (CIs) were reported.

All analyses were conducted using SAS version 9.4 (SAS Institute), with statistical significance defined as *p* < .05.

## RESULTS

### UPHP participants characteristics

Of the 199 potential identified cases, 183 (92.0%) were included in the analysis, and 16 were excluded. Excluded records included participants who completed an evaluation but did not sign a monitoring agreement (*n* = 5), preliminary records for individuals with multiple enrollments (*n* = 4), and cases with missing or unreadable documentation (*n* = 7). A total of 102 records were for individuals enrolled between 2013 and 2019 (*n* = 102) with complete 5‐year outcome data. An additional 81 records were for individuals enrolled in 2020 or later, which were included only in baseline analyses. Table 1 summarizes demographic and professional characteristics of the UPHP participants at the time of enrollment.

**Table 1:** Descriptive statistics of the UPHP participants: Total versus 5‐year follow‐up sample.

Variable	Total sample (*n* = 183)	5‐Year sample (*n* = 102)
	*n* (%)	*n* (%)
Age at enrollment (years), mean ± SD	40.5 ± 8.7	39.4 ± 8.8
Duration of SUD prior to PHP enrollment (years), mean ± SD	4.6 ± 5.7	5.3 ± 6.4
Gender		
Women	89 (48.6)	52 (51.0)
Men	94 (51.4)	50 (49.0)
Professions		
Nurse	111 (60.6)	59 (57.8)
Physician	33 (18.0)	18 (17.7)
Pharmacists and technicians	10 (5.5)	4 (3.9)
Dentist	7 (3.8)	6 (5.9)
“Other” professions	22 (12.0)	15 (14.7)
Primary substance used		
Opioids	86 (47.0)	51 (50.0)
Alcohol	74 (40.4)	35 (34.3)
Stimulants	6 (3.3)	0 (0.0)
Cannabis	6 (3.3)	0 (0.0)
Inhalants	4 (2.2)	0 (0.0)
Multiple substances	4 (2.2)	16 (15.7)
Benzodiazepines	3 (1.6)	0 (0.0)
Trauma history		
No trauma	70 (38.3)	32 (31.4)
Unknown	54 (29.5)	36 (35.3)
Emotional trauma	27 (14.8)	16 (15.7)
Domestic violence	11 (6.0)	8 (7.8)
Multiple trauma	11 (6.0)	5 (4.9)
Sexual Abuse	10 (5.5)	5 (4.9)
Psychiatric comorbidity		
No psychiatric diagnosis	33 (18.0)	20 (19.6)
Unknown	14 (7.7)	10 (9.8)
Mood disorders	34 (18.6)	21 (20.6)
Anxiety and trauma disorders	25 (13.7)	14 (13.7)
Multiple diagnoses	69 (37.7)	35 (34.3)
Neurodevelopmental disorders	6 (3.3)	2 (2.0)
Personality disorders	2 (1.1)	0 (0.0)
Injury or pain history		
No Injury	67 (36.6)	31 (30.4)
Unknown	41 (22.4)	26 (25.5)
Musculoskeletal injury	33 (18.0)	18 (17.7)
Postsurgical pain	21 (11.5)	16 (15.7)
Neurological pain	11 (6.0)	6 (5.9)
Internal conditions	6 (3.3)	2 (2.0)
Dental pain	4 (2.2)	3 (2.9)
Dilute or aberrant toxicology results		
No dilute/aberrant result	125 (68.3)	58 (56.9)
1 dilute/aberrant result	35 (19.1)	24 (23.5)
≥2 dilute/aberrant result	23 (12.6)	20 (19.6)

*Note*: Professions with fewer than five participants were grouped into the “Other” category to ensure adequate cell sizes for analysis. An exception was made for pharmacists in the 5‐year sample to maintain consistency with the profession categories presented in the total sample. (Total sample): Professions in “Other”: Dental Hygienists (*n* = 2), Physician Assistant (*n* = 4), Psychologists & Counselors (*n* = 4), Radiologic Technologist (*n* = 3), Recreational Therapists (*n* = 2), Social Workers (*n* = 3), and Veterinarians (*n* = 4). (5‐year sample): Professions in “Other”: Physician Assistant (*n* = 1), Psychologists & Counselors (*n* = 4), Radiologic Technologist (*n* = 3), Recreational Therapists (*n* = 2), Social Workers (*n* = 2), and Veterinarians (*n* = 3).

**Figure 1:**
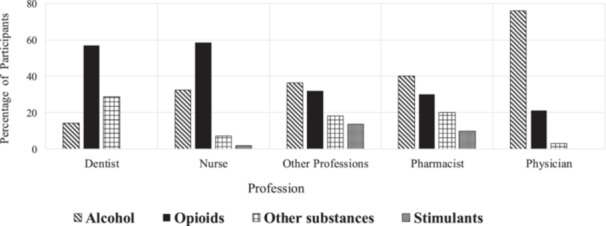
Distribution of primary substance used at enrollment across professions. Other Substances—Benzodiazepines, Cannabis, Inhalants, and Sedatives. Footnote b: Other Professions—Dental hygienists, Physician assistants, Psychologists, Radiologic technologists, Recreational therapists, social workers, and Veterinarians.

The cases were roughly split between men (51.4%) and women (48.6%), with mean enrollment age of 41 years. Nurses comprised the largest group (60.6%). Figure 1 displays the distribution of primary substances at enrollment, stratified by profession. Alcohol use predominated among physicians (approximately 76.0%), opioid use among nurses (59.0%) and dentists (57.0%) while pharmacists and “Other” professionals category demonstrated a more evenly distributed pattern. Stimulant use was infrequent across all groups.

Cases were distributed across 12 enrollment cohorts from 2013 to 2024, with the highest representation in the 2022 (*n* = 24, 13.1%) and lowest representation in 2014 (*n* = 8, 4.4%).

### Monitoring and treatment overview

Most UPHP participants (90.2%) signed a 5‐year monitoring agreement with shorter contracts reflecting transfers from other PHPs (9.8%). In such cases, UPHP generally honors the original PHP monitoring agreement parameters, provided the participant has not experienced a return to use or raised other significant concerns. The mean duration of SUD before UPHP enrollment was 4.6 ± 5.7 years (range: 1 month– 40 years). Multiple treatment types were recommended, with most participants engaging in more than one modality. Mutual support groups were the most frequently recommended intervention (84.2%), followed by intensive outpatient treatment (48.6%) and psychotherapy (43.2%). Inpatient/residential treatment was recommended for 28.3%, detoxification for 1.6%. Medication for addiction treatment (MAT) was used in 16.9% of opioid use disorder cases and 15.0% of alcohol use disorder cases, though specific medications were inconsistently recorded.

### Toxicology monitoring and return‐to‐use patterns

Nearly all participants (98%) underwent random urine drug screening. By the end of year one, 48.1% (*n* = 88) remained fully abstinent, while 19.1% (*n* = 35) tested positive for unapproved substances, and 17.5% (*n* = 32) showed aberrant results. Across all monitored cohorts (2013–2024), 46.5% (*n* = 85) experienced at least one return‐to‐use episode during their monitoring period, 49.2% (*n* = 90) did not, and 4.4% (*n* = 8) had an unknown status due to incomplete records. Return to use was most frequent in the first year (33.9%), with lower frequencies thereafter. The method of identification (toxicology vs. self‐report) was inconsistently recorded.

### Monitoring and occupational outcomes in 1st and 5th year

#### Monitoring status

At 1 year, 92.9% (*n* = 170) remained in monitoring, 2.2% (*n* = 4) transferred to another state PHP, and 4.9% (*n* = 9) were dismissed for nonadherence (e.g., multiple missed toxicology tests, poor attendance, or significant return to use). Among those with 5‐year follow‐up data, 81.4% (*n* = 83) successfully completed monitoring, 5.9% (*n* = 6) transferred to other PHPs, 2.9% (*n* = 3) extended monitoring, 7.8% (*n* = 8) were dismissed or left against UPHP advice, 2.0% (*n* = 2) participants died during monitoring.

#### Licensure status

At year one, 72.1% (*n* = 132) retained full licensure, 18.0% (*n* = 33) were practicing under restrictions (e.g., probation or controlled substance prescribing restrictions), 4.4% (*n* = 8) surrendered their licenses (disciplinary or voluntary), and 5.5% (*n* = 10) had not yet obtained licensure due to relocation or pending credentialing. By year 5 (2013–2019 cohort), 75.5% (*n* = 77) remained fully licensed, 11.8% (*n* = 12) had disciplinary surrenders, 9.8% (*n* = 10) had expired licenses (including retirements), and 2.9% (*n* = 3) were not applicable (primarily students at enrollment who later transitioned to full licensure).

#### Employment status

At year one, 42.6% (*n* = 78) worked full‐time in healthcare, 41.5% (*n* = 76) were unemployed, 3.8% (*n* = 7) worked part‐time in healthcare, 3.3% (*n* = 6) worked outside healthcare, and 2.7% (*n* = 5) had transitioned to another specialty. These included clinical residents who changed specialties (e.g., from anesthesiology to emergency medicine or family medicine) and nurses transitioning into non‐clinical or administrative roles away from bedside care. Employment status was unknown for 6.0% of participants (*n* = 11).

By year five, 55.9% (*n* = 57) were employed full‐time in healthcare, 2.0% (*n* = 2) part‐time in healthcare, 6.9% (*n* = 7) were employed outside healthcare, and 2.9% (*n* = 3) had transitioned to another specialty. Employment status was not recorded for 23.5% (*n* = 24) of participants, which included 8.8% (*n* = 9) who had no assigned fifth‐year occupational status due to early program completion or extended monitoring.

Exploratory analyses found no licensure association, but higher return to use among non‐employed participants (table not shown).

#### Completion rate across professions

While patterns of substance use varied across professional groups, completion rates of the UPHP monitoring program were notably consistent (see Table 2). Analysis was limited to the 2013–2019 cohort, with completion defined as successfully finishing monitoring (initial and/or extended) or transferring to another state program. All other outcomes were categorized as noncompletion.

**Table 2:** Program completion status stratified by profession.

Profession	Completed *n* (%)	Did Not complete *n* (%)	Total (*n*)
Dentist	6 (100.0%)	0 (0.0%)	6
Nurse	49 (83.1%)	10 (16.9%)	59
Other*	12 (80.0%)	3 (20.0%)	15
Pharmacist	4 (100.0%)	0 (0.0%)	4
Physician	15 (83.3%)	3 (16.7%)	18
Total	86 (84.4%)	16 (15.7%)	102

*Note*: Professions with fewer than four participants were grouped into the “Other*” category to ensure adequate cell sizes for analysis. Professions in “Other”: Physician Assistant (*n* = 1), Psychologists & Counselors (*n* = 4), Radiologic Technologist (*n* = 3), Recreational Therapists (*n* = 2), Social Workers (*n* = 2), and Veterinarians (*n* = 3).

Completion rates were high across all groups. Given small, expected counts, Fisher's test showed no statistically significant association between profession and completion status (two‐sided *p* = .888).

#### Predictors for return to use over the period of monitoring

A total of 41 out of 102 participants with 5‐year data (43.6%) experienced a return to use episode during the monitoring period. Logistic regression was used to examine demographic and clinical predictors of return to use (Table 3). Reference groups were male (gender), injury = yes (injury history), nurse (profession), and 0 dilute results (toxicology). For continuous predictors, odds ratios reflect change per 1‐year increase. In the adjusted model, older age at enrollment was associated with lower odds of return to use (OR = 0.933 per additional year, 95% CI: 0.874–0.996, *p* = .039), while longer pre‐monitoring duration of substance use was significantly associated with higher odds (OR = 1.141 per year increase, 95% CI: 1.044–1.249, *p* = .004). Participants without a documented injury history had lower odds of return to use (OR = 0.226, 95% CI: 0.067–0.767, *p* = .017). Participants with dilute urine toxicology results and those working in the nursing profession showed nonsignificant trends toward higher risk of return to use.

**Table 3:** Logistic regression predicting return to use at any point during monitoring with UPHP.

Predictor variable	Model 1 (unadjusted analysis)			Model 2 (adjusted analysis)		
	Odds ratio (Unadjusted)	95% CI	*p*‐value	Odds ratio (adjusted)	95% CI	*p*‐value
Age at enrollment	0.975	0.929–1.023	0.303	0.933	0.874–0.996	0.039*
Gender (female vs. male)	2.038	0.889–4.674	0.093	1.544	0.567–4.207	0.396
Duration of SUD pre‐UPHP	1.047	0.979–1.119	0.181	1.141	1.044–1.249	0.004**
Injury History						
No vs. yes	0.429	0.158–1.162	0.096	0.226	0.067–0.767	0.017*
Unknown vs. yes	0.806	0.294–2.212	0.675	0.667	0.214–2.073	0.484
Profession						
Physician vs. nurse	0.455	0.140–1.479	0.190	0.404	0.100–1.638	0.205
Other professions vs. nurse	0.571	0.207–1.576	0.280	0.549	0.164–1.832	0.329
Dilute or aberrant toxicology results						
1 dilute result vs. 0 dilute result	1.136	0.421–3.066	0.802	0.983	0.320–3.015	0.976
2 or more dilute results vs. 0 dilute result	1.640	0.570–4.721	0.359	3.027	0.812–11.292	0.099

*Note*: Other professions—Combination of all other health professions in the sample | Other substances— Combination of all other substances in the sample.

Abbreviation: CI, confidence interval.

*p < .05, **p < .01.

## DISCUSSION

This 11‐year retrospective study offers insight into the recovery trajectory of nurses and other HCPs in the UPHP. The program's structure aligns with national best practices from the FSPHP, including comprehensive evaluation, long‐term monitoring, and structured support.^11^ Overall, important differences emerged in this nurse and allied health professional‐dominant sample compared to prior studies of physician‐only outcomes.

Return to use was most common in the first year (33.9%), highlighting early recovery vulnerability.^19^ However, return to use rates in this cohort remained relatively stable between years 2 and 5, reinforcing the need for sustained intensive support and oversight throughout the monitoring period. While the overall return to use rate (48.6%) was higher than physician‐only samples, the completion rate (81.4%) was comparable to 75%–95% success rates seen in prior physician‐only studies.^12^, ^15^, ^20^ These findings likely reflect both the strengths of UPHP's monitoring model and the distinct structural and occupational factors faced by this predominantly non‐physician cohort. McLellan et al.,^15^ found that approximately 81% of PHP participants had no recurrence of substance use over 5 years.^15^ However, their cohort consisted entirely of physicians, most of whom received extended residential care followed by structured outpatient support; whereas, the present study primarily involved nurses and allied health professionals with more limited access to intensive treatment.

Residential treatment was recommended for some, but outcomes were not analyzed by treatment intensity. Uptake of MAT, including medication for opioid use disorder, was also low, despite strong evidence of its effectiveness in reducing opioid use, preventing overdose, and improving treatment retention in the general population.^21^ UPHP supports the use of MAT for all professionals, with treatment decisions made collaboratively between participants, treatment providers, and UPHP. There were no formal or profession‐specific restrictions, and treatment providers/centers generally supported MAT when clinically indicated. Thus, the limited MAT utilization may reflect participant preferences, prescribing practices, and/or program‐level or employer‐driven barriers and warrants further investigation. Exploring whether treatment intensity and/or use of MAT influence return to use or program completion in a larger sample could offer actionable insights. In addition, structural factors like licensing and workplace protections, may affect monitoring engagement or sustained PHP participation. Nurses, particularly, face stricter licensing sanctions and fewer protections, which can shape treatment participation and recovery outcomes.^22^, ^23^

By year 5, 55.9% of UPHP participant records documented full‐time employment in healthcare. In comparison, physician‐only studies reported higher reentry rates, with McLellan et al. noting that 78.7% of physicians were licensed and practicing at 5‐year follow‐up, and Skipper et al. reporting continuation rates of 76% among anesthesiologists and 73% among other physicians.^15^, ^24^ However, in the current study, 23.5% had unknown employment status, limiting conclusions. Inconsistencies in how employment was recorded by URAP/UPHP and during data abstraction may have contributed to underestimation. Since at least 16 h per week of professional or related work are required for successful completion of UPHP monitoring, actual employment rates were likely higher than reported here. These preliminary differences suggest profession‐specific barriers to reentering clinical practice among non‐physicians. Barriers to reemployment may include stigma, delays in licensure decisions, financial pressures, and limited workplace understanding of SUD.^25^, ^26^ Recent findings among nurse anesthetists further underscore these challenges, highlighting persistent stigma and structural obstacles to practice reentry following SUD treatment.^27^ These disparities suggest that nurses and allied professionals may be treated differently than physicians, pointing to an important area for future study and intervention. Targeted reintegration efforts are needed to address the unique experiences of non‐physician professionals. Additionally, some research links unemployment with poorer recovery outcomes and increased risk of return to use risk, reinforcing the value of career stability in recovery.^28^

Licensure outcomes add further context. By year 5, 75.5% of cases retained active licensure, while only about 12% had surrendered their licenses due to disciplinary measures. These outcomes are consistent with McLellan et al., which reported a 14% loss of licensure rate among monitored physicians. Some license surrenders likely reflect career transitions, but others may indicate difficulty sustaining recovery, underscoring the need for continued program engagement.^15^

In the final multivariable model, return to use was significantly associated with age, longer duration of addiction prior to UPHP enrollment, and a documented history of injury or pain. Participants with an injury history had higher odds of return to use, which may indicate a more complex clinical presentation. For some, substance use may have originated in the context of managing chronic pain or injury‐related conditions, potentially involving long‐term prescriptions or unresolved physical discomfort. This pathway may contribute to greater difficulty sustaining abstinence during monitoring. These findings underscore the importance of tailoring support strategies for individuals with injury‐related substance use histories and highlight the need for integrated pain and addiction treatment approaches in professional health programs. Recent research has shown that HCPs may be uniquely vulnerable to pain‐related craving and disruptions in craving and abstinence self‐efficacy during recovery, reinforcing the relevance of addressing pain as part of treatment planning.^29^ No statistically significant associations were found for profession, gender, or dilute/aberrant toxicology results, suggesting that treatment history may be a more meaningful predictor of return to use than demographic traits or testing aberrancies.

### Implications and limitations

These findings highlight the importance of strengthening early‐phase recovery support, particularly during the first year when return to use is most common. Structured peer mentorship and individualized care integrating mental health and pain management may improve outcomes. Although MAT use was limited, UPHP supports its application across professions, and provider education, clear treatment protocols, and stigma reduction could enhance acceptance. Tailored reintegration efforts, licensure advocacy, workplace education, and career counseling may further support reentry, especially for non‐physician professionals.

This study's strengths include its 11‐year scope and use of real‐world clinical data. However, findings are limited by reliance on retrospective records, incomplete documentation, and the single‐state design, which may limit generalizability. Because return to use was recorded a binary event per participant, a logistic regression was used, instead of grouped time‐to‐event survival analyses for ease of interpretation; however, this approach resulted in the use of fewer data. Incomplete substance‐specific data also limited exploration of associations between treatment types and outcomes. Future prospective studies may help capture contextual factors influencing recovery.

## CONCLUSION AND FUTURE DIRECTIONS

This retrospective analysis highlights recovery and professional outcomes among HCPs monitored through the UPHP. Return to use was most common in the first year of monitoring, underscoring the need for intensive early support. Although psychiatric comorbidities were not predictive of return to use, their high prevalence highlights the importance of integrated mental health services throughout monitoring. Despite high completion and licensure rates, reemployment challenges, especially among non‐physician professionals, remain significant. Integrating peer coaching, licensure navigation, and career reintegration may enhance outcomes. Future research should examine how specific interventions influence long‐term outcomes and support sustainable recovery across healthcare professions. Larger multisite cohorts are also needed to enhance generalizability and inform best practices for supporting healthcare professionals in recovery.

## CONFLICT OF INTEREST STATEMENT

The authors declare that there are no conflict of interests.

## References

[1] Wasmuth S , Pritchard K , Kaneshiro K . Occupation‐based intervention for addictive disorders: a systematic review. J Subst Abuse Treat. 2016;62:1‐9. 10.1016/j.jsat.2015.11.011 26738639

[2] Kumar S . Burnout and doctors: prevalence, prevention and intervention. Healthcare. 2016;4(3):37. 10.3390/healthcare4030037 27417625 PMC5041038

[3] Lander L , Howsare J , Byrne M . The impact of substance use disorders on families and children: from theory to practice. Soc Work Public Health. 2013;28(3‐4):194‐205. 10.1080/19371918.2013.759005 23731414 PMC3725219

[4] Oyefeso A , Clancy C , Farmer R . Prevalence and associated factors in burnout and psychological morbidity among substance misuse professionals. BMC Health Serv Res. 2008;8:39. 10.1186/1472-6963-8-39 18261227 PMC2265695

[5] Shadakshari D , Muliyala KP , Jayarajan D , Kandasamy A . Occupational challenges in physicians with substance use disorder: a qualitative study. Indian J Psychol Med. 2022;44(3):253‐258. 10.1177/02537176211020520 35656432 PMC9125475

[6] Stone L . Addressing substance use disorders: implications for nursing and for health care systems. Nurs Clin North Am. 2023;58(2):xiii‐xiv. 10.1016/j.cnur.2023.02.005 37105661

[7] Bennett J , OʼDonovan D . Substance misuse by doctors, nurses and other healthcare workers. Curr Opin Psychiatry. 2001;14(3):195‐199. 10.1097/00001504-200105000-00006

[8] Kunyk D . Substance use disorders among registered nurses: prevalence, risks and perceptions in a disciplinary jurisdiction. J Nurs Manag. 2015;23(1):54‐64. 10.1111/jonm.12081 23952722

[9] Baldisseri MR . Impaired healthcare professional. Crit Care Med. 2007;35(2 suppl):S106‐S116. 10.1097/01.Ccm.0000252918.87746.96 17242598

[10] Shaw MF , McGovern MP , Angres DH , Rawal P . Physicians and nurses with substance use disorders. J Adv Nurs. 2004;47(5):561‐571. 10.1111/j.1365-2648.2004.03133.x 15312119

[11] Federation of State Physician Health Programs (FSPHP). Mission, vision, and values. Accessed March 5, 2025, 2025. https://www.fsphp.org/#:~:text=Mission%2C%20Vision%20and%20Values,and%20the%20patients%20they%20serve.

[12] DuPont RL , McLellan AT , White WL , Merlo LJ , Gold MS . Setting the standard for recovery: physicians' health programs. J Subst Abuse Treat. 2009;36(2):159‐171. 10.1016/j.jsat.2008.01.004 19161896

[13] Utah Professionals Health Program. Monitoring and Advocacy Accessed March 5, 2025, 2025. https://uphp.utah.gov/monitoring-advocacy/

[14] Smiley R , Reneau K . Outcomes of substance use disorder monitoring programs for nurses. J Nurs Regul. 2020;11(2):28‐35. 10.1016/S2155-8256(20)30107-1

[15] McLellan AT , Skipper GS , Campbell M , DuPont RL . Five year outcomes in a cohort study of physicians treated for substance use disorders in the United States. BMJ. 2008;337:a2038. 10.1136/bmj.a2038 18984632 PMC2590904

[16] American Medical Association. *Access to confidential health services for medical students and physicians*. 2019. BOT Report 15, A‐19. Accessed June 24, 2025. https://www.ama-assn.org/system/files/2019-04/a19-bot15.pdf

[17] Harris PA , Taylor R , Thielke R , Payne J , Gonzalez N , Conde JG . Research electronic data capture (REDCap)—a metadata‐driven methodology and workflow process for providing translational research informatics support. J Biomed Inf. 2009;42(2):377‐381. 10.1016/j.jbi.2008.08.010 PMC2700030 18929686

[18] Harris PA , Taylor R , Minor BL , et al. The REDCap consortium: building an international community of software platform partners. J Biomed Inf. 2019;95:103208. 10.1016/j.jbi.2019.103208 PMC7254481 31078660

[19] Brandon TH , Vidrine JI , Litvin EB . Relapse and relapse prevention. Annu Rev Clin Psychol. 2007;3(1):257‐284. 10.1146/annurev.clinpsy.3.022806.091455 17716056

[20] Buhl A . Prognosis for the recovery of surgeons from chemical dependency: a 5‐year outcome study. AArch Surg. 2011;146(11):1286‐1291. 10.1001/archsurg.2011.271 22106321

[21] Volkow ND , Frieden TR , Hyde PS , Cha SS . Medication‐assisted therapies—tackling the opioid‐overdose epidemic. N Engl J Med. 2014;370(22):2063‐2066. 10.1056/nejmp1402780 24758595

[22] Foong‐Reichert A‐L , Fung A , Carter CA , Grindrod KA , Houle SKD . Characteristics, predictors and reasons for regulatory body disciplinary action in healthcare: a scoping review. Journal of Medical Regulation. 2021;107(4):17‐31. 10.30770/2572-1852-107.4.17

[23] Zhong EH , Kenward K , Sheets VR , Doherty ME , Gross L . Original research: probation and recidivism remediation among disciplined nurses in six states. Am J Nurs. 2009;109(3):48‐57. 10.1097/01.naj.0000346931.36111.e9 19240497

[24] Skipper GE , Campbell MD , DuPont RL . Anesthesiologists with substance use disorders: a 5‐year outcome study from 16 state physician health programs. Anesth Analg. 2009;109(3):891‐896. 10.1213/ane.0b013e3181adc39d 19690263

[25] Cook LM . Can nurses trust nurses in recovery reentering the workplace? Nursing. 2013;43(3):21‐24. 10.1097/01.nurse.0000427092.87990.86 23411547

[26] Matthias‐Anderson D , Yurkovich E . Work reentry for RNs after substance use disorder treatment: implications for the nursing profession. J Nurs Regul. 2016;7(3):26‐32. 10.1016/s2155-8256(16)32318-3

[27] Carter T , Wingo N , McMullan S , et al. Challenges for nurse anesthetists reentering practice following substance use disorder treatment. presented at: Aana j; Apr 2024; Accessed June 30, 2025. 38564209

[28] Henkel D . Unemployment and substance use: a review of the literature (1990‐2010). Curr Drug Abuse Rev. 2011;4(1):4‐27. 10.2174/1874473711104010004 21466502

[29] Lysandrou AE , Teitelbaum SA , Merlo L , et al. Co‐occurring pain and addiction: prognostic implications for healthcare professionals in residential treatment for substance use disorder. J Addict Dis. 2024;42(4):335‐344. 10.1080/10550887.2023.2223505 37380371

